# Supplementary material for: ADAR1 p150 prevents HSV-1 from triggering PKR/eIF2α-mediated translational arrest and is required for efficient viral replication
Source: PLoS Pathog. 2025 Apr 8;21(4):e1012452. doi: 10.1371/journal.ppat.1012452 (PMC12011305; doi:10.1371/journal.ppat.1012452)
Supplement: S1 Fig — a) Transcripts ENSG00000160710 (ADAR1) and ENSG00000197381 (ADARB1/ADAR2) indicated as reads per kilobase per million mapped reads (RPKM) taken from total RNA of HFF infected with HSV-1 strain 17 at MOI 10 (Rutkowski et al., 2015)[75]. b) WT were infected with HSV-1 at MOI=1. After 1 hr infectious media was replaced with fresh media. One set was treated with 10ng/mL human IFNb for 24h. Cells were collected in TRIreagent at indicated times after infection (h.p.i.). RNA was extracted and RT-qPCR was performed on indicated genes. All samples were normalized to Mock18S and expressed as relative expression to Mock. Data is shown as mean ± standard deviation (SD); *, p≤0.05 denoted only for relative expression >1, by One-Way ANOVA. (DOCX) [file ppat.1012452.s001.docx]

**S1 Fig. Levels of ADAR1 and ADAR2 transcripts decrease during productive HSV-1 infection**

a. dataset from (Rutkowski et al., 2015)[75]

A) ADAR1 B) ADAR2

b. relative expression levels of ADAR1p110, ADAR1p150 and ADAR2 in HEK293 WT cells

**S1 Fig. Levels of ADAR1 and ADAR2 transcripts decrease during productive HSV-1 infection. a)** Transcripts ENSG00000160710 (ADAR1) and ENSG00000197381 (ADARB1/ADAR2) indicated as reads per kilobase per million mapped reads (RPKM) taken from total RNA of HFF infected with HSV-1 strain 17 at MOI 10 (Rutkowski et al., 2015)[75]. **b)** WT were infected with HSV-1 at MOI=1. After 1 hr infectious media was replaced with fresh media. One set was treated with 10ng/mL human IFNβ for 24h. Cells were collected in TRIreagent at indicated times after infection (hpi). RNA was extracted and RT-qPCR was performed on indicated genes. All samples were normalized to Mock18S and expressed as relative expression to Mock. Data is shown as mean ± standard deviation (SD); *, p≤0.05 denoted only for relative expression >1, by One-Way ANOVA.
