# Supplementary material for: ADAR1 p150 prevents HSV-1 from triggering PKR/eIF2α-mediated translational arrest and is required for efficient viral replication
Source: PLoS Pathog. 2025 Apr 8;21(4):e1012452. doi: 10.1371/journal.ppat.1012452 (PMC12011305; doi:10.1371/journal.ppat.1012452)
Supplement: S2 Fig — WT and ADAR1 KO cells were seeded in 48 well plate. and treated with 2µM Staurosporin (STR) or 20µM zVAD (ZVD) or both for 3hours. 0.5% DMSO were used as control. Levels of apoptosis were tested with Caspase-glo assay (Caspase-Glo 3/7 assay, Promega) as per manufacturer’s protocol. Data is shown as mean ± standard deviation (SD); ns – not statistically significant; Student’s t test independently performed for each denoted pair. (DOCX) [file ppat.1012452.s002.docx]

**S2 Fig. Pan-caspase inhibitor zVAD does not enhance HSV-1 replication in ADAR1 deficient cells**

**S2 Fig. Pan-caspase inhibitor zVAD does not enhance HSV-1 replication in ADAR1 deficient cells.** WT and ADAR1 KO cells were seeded in 48 well plate. and treated with 2µM Staurosporin (STR) or 20µM zVAD (ZVD) or both for 3hours. 0.5% DMSO were used as control. Levels of apoptosis were tested with Caspase-glo assay (Caspase-Glo 3/7 assay, Promega) as per manufacturer's protocol. Data is shown as mean ± standard deviation (SD); ns – not statistically significant; Student’s t test independently performed for each denoted pair.
