# Supplementary material for: ADAR1 p150 prevents HSV-1 from triggering PKR/eIF2α-mediated translational arrest and is required for efficient viral replication
Source: PLoS Pathog. 2025 Apr 8;21(4):e1012452. doi: 10.1371/journal.ppat.1012452 (PMC12011305; doi:10.1371/journal.ppat.1012452)
Supplement: S3 Fig — Cells were transfected with indicated siRNA. After 24 hours cells were infected with HSV-1 (MOI=1). a) At 24h.p.i. supernatant was collected for plaque assay. b) At 24h.p.i. cells were collected in RIPA for western blot. Data is shown as mean ± standard deviation (SD); not statistically significant (not shown); ****, p<0.0001, by One-Way ANOVA for (a) (DOCX) [file ppat.1012452.s003.docx]

**S3 Fig. PKR depletion rescues HSV-1 replication in A549 cells depleted of ADAR1**

a. siRNA screen in A549

b. WB showing downregulation of ADAR expression


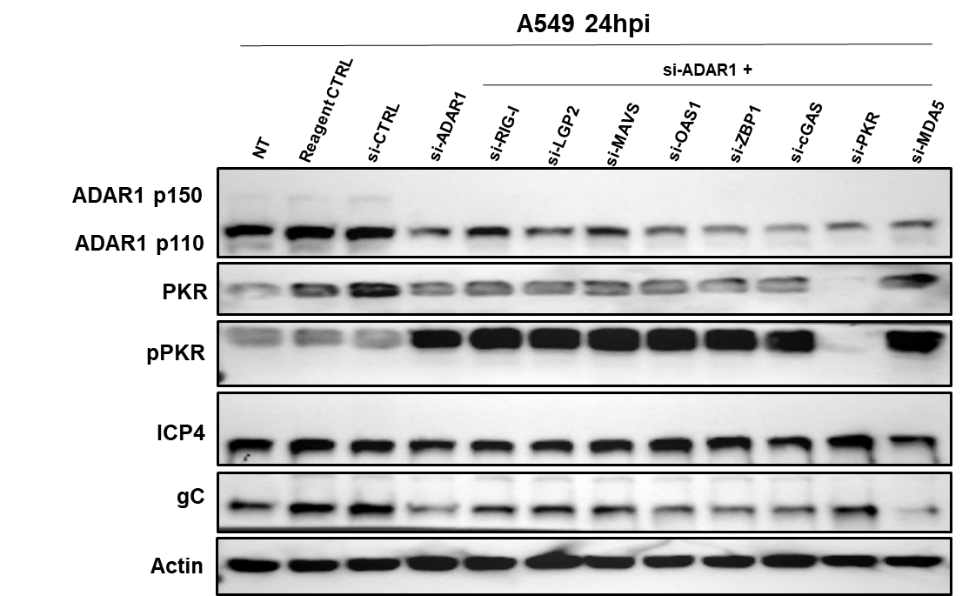


**S3 Fig. PKR depletion rescues HSV-1 replication in A549 cells depleted of ADAR1.** Cells were transfected with indicated siRNA. After 24 hours cells were infected with HSV-1 (MOI=1). **a)** At 24hpi supernatant was collected for plaque assay. **b)** At 24hpi cells were collected in RIPA for western blot. Data is shown as mean ± standard deviation (SD); not statistically significant (not shown); ****, p<0.0001 , by One-Way ANOVA for **(a)**
