# Supplementary material for: ADAR1 p150 prevents HSV-1 from triggering PKR/eIF2α-mediated translational arrest and is required for efficient viral replication
Source: PLoS Pathog. 2025 Apr 8;21(4):e1012452. doi: 10.1371/journal.ppat.1012452 (PMC12011305; doi:10.1371/journal.ppat.1012452)
Supplement: S4 Fig — a) Transcripts ENSG00000128272 (ATF4) and ENSG00000111640 (GAPDH) are indicated as reads per kilobase per million mapped reads (RPKM) taken from total RNA of HFF cells infected with HSV-1 strain 17 at MOI 10 (Rutkowski et al., 2015). b) HEK293A WT and ADAR1 KO were infected with HSV-1 at MOI=1. After 1 hr infectious media was replaced with fresh media. Cells were collected in TRIreagent at indicated timepoints (h.p.i.). RNA was extracted and RT-qPCR was performed on indicated genes. All samples were normalized to Mock18S and expressed as relative expression to Mock. Experiment was performed in two independent replicates. (DOCX) [file ppat.1012452.s004.docx]

**S4 Fig. Levels of ATF-4 mRNA durig productive HSV-1 infection**

a. dataset from (Rutkowski et al., 2015)

A) ATF-4 B) GAPDH

b. Expression pattern of ATF-4 in HEK293A WT and ADAR1 KO upon HSV-1 infection (MOI=0.5)

**S4 Fig. Levels of ATF-4 mRNA durig productive HSV-1 infection. a)** Transcripts ENSG00000128272 (ATF4) and ENSG00000111640 (GAPDH) are indicated as reads per kilobase per million mapped reads (RPKM) taken from total RNA of HFF cells infected with HSV-1 strain 17 at MOI 10 (Rutkowski et al., 2015). **b)** HEK293A WT and ADAR1 KO were infected with HSV-1 at MOI=1. After 1 hr infectious media was replaced with fresh media. Cells were collected in TRIreagent at indicated timepoints (hpi). RNA was extracted and RT-qPCR was performed on indicated genes. All samples were normalized to Mock18S and expressed as relative expression to Mock. Experiment was performed in two independent replicates.
