# Supplementary material for: ADAR1 p150 prevents HSV-1 from triggering PKR/eIF2α-mediated translational arrest and is required for efficient viral replication
Source: PLoS Pathog. 2025 Apr 8;21(4):e1012452. doi: 10.1371/journal.ppat.1012452 (PMC12011305; doi:10.1371/journal.ppat.1012452)
Supplement: S5 Fig — (a-b) Cells were transfected with indicated siRNA. After 24 hours cells were infected with HSV-1 (MOI=3). a) At given timepoints (h.p.i.) cells were collected in RIPA buffer for Western blot. b) At 24h.p.i. supernatants were collected for plaque assay. Data is shown as mean ± standard deviation (SD); ns – not statistically significant; by One-Way ANOVA. (DOCX) [file ppat.1012452.s005.docx]

**S5 Fig. Depletion of ADAR1p150 limits HSV-1 infection in A549 cells**

b. Plaque assay

a. Western blot


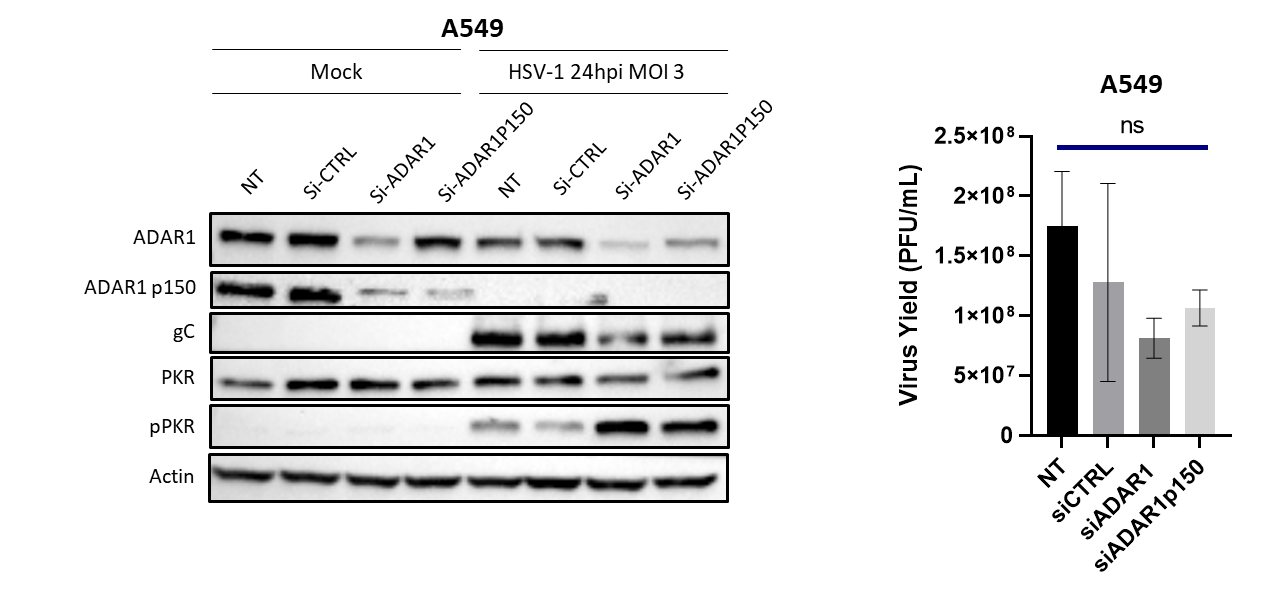


**S5 Fig. Depletion of ADAR1p150 limits HSV-1 infection in A549 cells. (a-b)** Cells were transfected with indicated siRNA. After 24 hours cells were infected with HSV-1 (MOI=3). **a)** At given timepoints (hpi) cells were collected in RIPA buffer for western blot. **b)** At 24hpi supernatants were collected for plaque assay. Data is shown as mean ± standard deviation (SD); ns – not statistically significant; by One-Way ANOVA.
