# Supplementary material for: ADAR1 p150 prevents HSV-1 from triggering PKR/eIF2α-mediated translational arrest and is required for efficient viral replication
Source: PLoS Pathog. 2025 Apr 8;21(4):e1012452. doi: 10.1371/journal.ppat.1012452 (PMC12011305; doi:10.1371/journal.ppat.1012452)
Supplement: S6 Fig — HEK293 ADAR1 WT and KO cells were seeded in a 24 well plate and infected with MOI 3 for 7 and 12h and were treated with RNase A (Promega) (20ug/mL) and Shortcut RNase III (NEB) (2U/mL) each for 1 hour. At indicated h.p.i. cells were fixed, stained with J2 antibody and DAPI. a) Cells were imaged using Axio Observer Z1 fluorescence microscope. Mean fluorescence intensity (MFI) of 33 to 49 cells per group was measured using ZEN software (Carl Zeiss). b) Confocal images were captured from the microscopy slides in a) using the LSM880 confocal microscope (Carl Zeiss). Maximum intensity projection images made by overlap of sequential z-stacks in ZEN software (Carl Zeiss) are shown. Data is shown as mean ± standard deviation (SD); not statistically significant (not shown); ****, p≤0.0001, by One-Way ANOVA for (a) (DOCX) [file ppat.1012452.s006.docx]

**S6 Fig.** **dsRNAs levels increase in ADAR1 WT and KO cells infected with HSV-1**

a. Quantitative measurement in fluorescence microscopy

b. Representative confocal imaging


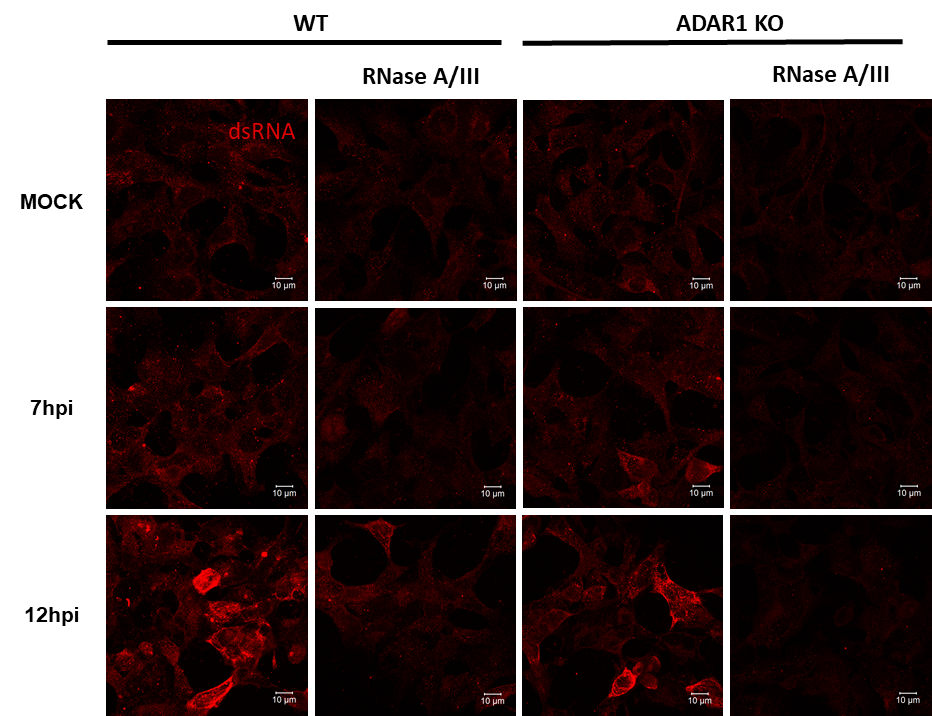


**S6 Fig.** **dsRNAs levels increase in ADAR1 WT and KO cells infected with HSV-1.** HEK293 ADAR1 WT and KO cells were seeded in a 24 well plate and infected with MOI 3 for 7 and 12h and were treated with RNase A (Promega) (20ug/mL) and Shortcut RNase III (NEB) (2U/mL) each for 1 hour. At indicated hpi cells were fixed, stained with J2 antibody and DAPI. **a)** Cells were imaged using Axio Observer Z1 fluorescence microscope. Mean fluorescence intensity (MFI) of 33 to 49 cells per group was measured using ZEN software (Carl Zeiss). **b)** Confocal images were captured from the microscopy slides in a) using the LSM880 confocal microscope (Carl Zeiss). Maximum intensity projection images made by overlap of sequential z-stacks in ZEN software (Carl Zeiss) are shown. Data is shown as mean ± standard deviation (SD); not statistically significant (not shown); ****, p≤0.0001, by One-Way ANOVA for **(a)**
