# Supplementary material for: ADAR1 p150 prevents HSV-1 from triggering PKR/eIF2α-mediated translational arrest and is required for efficient viral replication
Source: PLoS Pathog. 2025 Apr 8;21(4):e1012452. doi: 10.1371/journal.ppat.1012452 (PMC12011305; doi:10.1371/journal.ppat.1012452)
Supplement: S7 Fig — WT and ADAR1 KO were infected with HSV-1 (MOI=3). At 7h.p.i., cells were collected either in TRIreagent and total RNA was extracted. a) Total RNA blotted on charged membrane with positive and negative control. (LEFT) Image captured with HRP linked secondary. (RIGHT) Colorimetric image. b) ImageJ quantification of dot blot (n=18). Data is shown as mean ± standard deviation (SD); ‘ns’ not statistically significant, by One-Way ANOVA for (b) (DOCX) [file ppat.1012452.s007.docx]

**S7 Fig. Detection of total dsRNA using dot-blot**

a. dot-blot


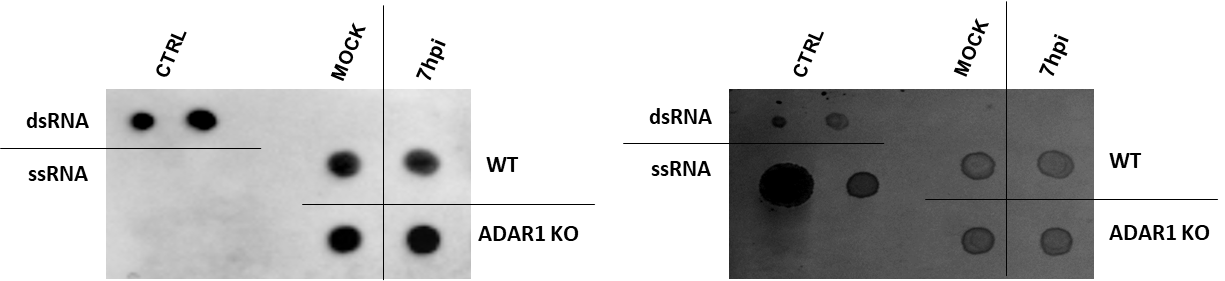


b. Quantification with ImageJ

**S7 Fig. Detection of total dsRNA using dot-blot.** WT and ADAR1 KO were infected with HSV-1 (MOI=3). At 7hpi, cells were collected either in TRIreagent and total RNA was extracted. **a)** Total RNA blotted on charged membrane with positive and negative control. (LEFT) Image captured with HRP linked secondary. (RIGHT) Colorimetric image. **b)** ImageJ quantification of dot blot (n=18). Data is shown as mean ± standard deviation (SD); 'ns' not statistically significant, by One-Way ANOVA for (b)
