# Supplementary material for: ADAR1 p150 prevents HSV-1 from triggering PKR/eIF2α-mediated translational arrest and is required for efficient viral replication
Source: PLoS Pathog. 2025 Apr 8;21(4):e1012452. doi: 10.1371/journal.ppat.1012452 (PMC12011305; doi:10.1371/journal.ppat.1012452)
Supplement: S8 Fig — HFF cells were seeded in a 24 well plate and infected with indicated MOI. Additionally, the cells infected with MOI 10 were treated with RNase A (Promega) (20ug/mL) and Shortcut RNase III (NEB) (2U/mL) each for 1 hour. At 7h.p.i. cells were fixed, stained with J2 antibody and DAPI or collected in RIPA. b) Cells were imaged using Axio Observer Z1 fluorescence microscope. Mean fluorescence intensity (MFI) of 21 to 26 cells per group was measured using ZEN software (Carl Zeiss). a) Confocal images were captured from the microscopy slides in a) using the LSM880 confocal microscope (Carl Zeiss). Maximum intensity projection images made by overlap of sequential z-stacks in ZEN software (Carl Zeiss) are shown. c) Western blot for viral proteins confirming infection in each MOI group. Data is shown as mean ± standard deviation (SD); not statistically significant (not shown); *, p≤0.5, by One-Way ANOVA for (b) (DOCX) [file ppat.1012452.s008.docx]

**S8 Fig.** **The level of dsRNAs is dependent on the MOI in HFFs infected with HSV-1**

a. Representative confocal imaging b. Quantitative measurement

c. Confirmation of infection

**
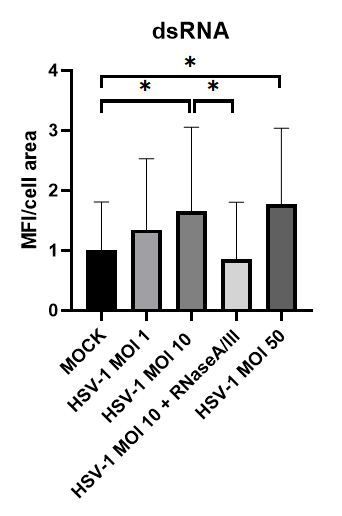
**

**S8 Fig.** **The level of dsRNAs is dependent on the MOI in HFFs infected with HSV-1**. HFF cells were seeded in a 24 well plate and infected with indicated MOI. Additionally, the cells infected with MOI 10 were treated with RNase A (Promega) (20ug/mL) and Shortcut RNase III (NEB) (2U/mL) each for 1 hour. At 7hpi cells were fixed, stained with J2 antibody and DAPI or collected in RIPA. **b)** Cells were imaged using Axio Observer Z1 fluorescence microscope. Mean fluorescence intensity (MFI) of 21 to 26 cells per group was measured using ZEN software (Carl Zeiss). **a)** Confocal images were captured from the microscopy slides in a) using the LSM880 confocal microscope (Carl Zeiss). Maximum intensity projection images made by overlap of sequential z-stacks in ZEN software (Carl Zeiss) are shown. **c)** Western blot for viral proteins confirming infection in each MOI group. Data is shown as mean ± standard deviation (SD); not statistically significant (not shown); *, p≤0.5, by One-Way ANOVA for **(b)**
