# Supplementary material for: ADAR1 p150 prevents HSV-1 from triggering PKR/eIF2α-mediated translational arrest and is required for efficient viral replication
Source: PLoS Pathog. 2025 Apr 8;21(4):e1012452. doi: 10.1371/journal.ppat.1012452 (PMC12011305; doi:10.1371/journal.ppat.1012452)
Supplement: S1 Table — (DOCX) [file ppat.1012452.s009.docx]

**S1 Table. List of siRNAs**

| **Sr. No.** | **siRNA** | **Manufacture#REF** |
| --- | --- | --- |
| 1 | RIG-I | Santa Cruz # sc-61480 |
| 2 | LGP-2 | Santa Cruz # sc-93967 |
| 3 | MAVS | Santa Cruz # sc-75755 |
| 4 | OAS-1 | Santa Cruz # sc-61241 |
| 5 | ZBP-1 | Santa Cruz # sc-61823 |
| 6 | cGAS | Santa Cruz # sc-95512 |
| 7 | MDA5 | Santa Cruz # sc-61010 |
| 8 | PKR | Santa Cruz # sc-36263 |
| 9 | ADAR-1 | Invitrogen # 4390824 s1007 |
| 10 | CTRL | Invitrogen # 4390843 |
| 11 | ADAR-1 p150 | Kind gift from J. Maelfeit |
