# Supplementary material for: ADAR1 p150 prevents HSV-1 from triggering PKR/eIF2α-mediated translational arrest and is required for efficient viral replication
Source: PLoS Pathog. 2025 Apr 8;21(4):e1012452. doi: 10.1371/journal.ppat.1012452 (PMC12011305; doi:10.1371/journal.ppat.1012452)
Supplement: S2 Table — (DOCX) [file ppat.1012452.s010.docx]

**S2 Table. List of antibodies**

| **Sr.No** | **Antibody** | **kDa** | **Host** | **Manufacturer#REF** | **Dilution for WB** |
| --- | --- | --- | --- | --- | --- |
| **1** | ADAR1 | 110, 150 | rabbit | Cell Signalling#14175S | 1:1000 |
| **2** | ADAR p150 | 150 | rabbit | Cell Signalling#32136S | 1:1000 |
| **3** | EIF2α | 38 | rabbit | Cell Signalling#9722S | 1:1000 |
| **4** | Phospho EIF2α (Ser51) | 38 | rabbit | Cell Signalling#3398S | 1:1000 |
| **5** | NFκB p65 | 65 | Rabbit | Santa Cruz Biotech#sc-372 | 1:1000 |
| **6** | Phospho -RELA/NFκB p65 (27.Ser 536) | 65 | Mouse | Santa Cruz Biotech#sc-136548 | 1:1000 |
| **7** | MAVS | 75,52 | rabbit | Cell Signalling#3993T | 1:1000 |
| **8** | MDA5 | 130 | rabbit | Cell Signalling#5321T | 1:500 |
| **9** | OAS1 | 40,44 | mouse | Santa Cruz Biotech#sc-515518 | 1:500 |
| **10** | PKR | 74 | rabbit | Cell Signalling#12297S | 1:1000 |
| **11** | Phospho PKR (T446) | 68 | rabbit | Abcam#ab32036 | 1:1000 |
| **12** | ATF-4 | 49 | rabbit | Cell Signalling#11815S | 1:500 |
| **13** | gC | 98 | mouse | Abcam#ab6509 | 1:2000 |
| **14** | ICP4 | 175 | mouse | Abcam#ab6514 | 1:2000 |
| **15** | ICP27 | 63 | mouse | Santa Cruz Biotech#sc-69807 | 1:2000 |
| **16** | TK | 42 | mouse | Pan et al | 1:2000 |
| **17** | ICP8 | 128 | mouse | Abcam#ab20194 | 1:2000 |
| **18** | ICP0 | 120 | mouse | Abcam#ab6513 | 1:2000 |
| **19** | VP16 | 58 | mouse | Abcam#ab110226 | 1:2000 |
| **20** | Actin | 42 | mouse | Merck#MAB1501 | 1:10000 |
| **21** | GFP | 26 | mouse | Santa Cruz Biotech#sc-9996 | 1:2000 |
| **22** | FLAG-M2 | - | rabbit | Cell Signalling#1479S | 1:2000 |
| **23** | Anti-Rabbit IgG HRP Linked Secondary | - | Goat | Cell Signalling#7074S | 1:2000 |
| **24** | Anti-Mouse IgG HRP Linked Secondary | - | horse | Cell Signalling#7076S | 1:2000 |
