# Supplementary material for: ADAR1 p150 prevents HSV-1 from triggering PKR/eIF2α-mediated translational arrest and is required for efficient viral replication
Source: PLoS Pathog. 2025 Apr 8;21(4):e1012452. doi: 10.1371/journal.ppat.1012452 (PMC12011305; doi:10.1371/journal.ppat.1012452)
Supplement: S3 Table — (DOCX) [file ppat.1012452.s011.docx]

**S3 Table. List of Primers**

| **Sr.No.** | **Gene** | **Organism** | **FORWARD** | **REVERSE** | **Source (REF)** |
| --- | --- | --- | --- | --- | --- |
| 1 | 18S | Human | GTAACCCGTTGSSCCCCATT | CCATCCAATCGGTAGTAGCG | [1, 5] |
| 2 | ATF-4 | Human | TTCTCCAGCGACAAGGCTAAGG | CTCCAACATCCAATCTGTCCCG | Origene#HP205494 |
| 3 | ICP4 | HSV-1 | TCGAGAGTCCGTAGGTGAC | TTGTTCTCCGACGCCATC | [4] |
| 4 | ICP0 | HSV-1 | AGCGAGTACCCGCCGGCCTG | CAGGTCTCGGTCGCAGGGAAAC | [4] |
| 5 | ICP27 | HSV-1 | GTGTGCAGCCGTGTTCCAA | AGCGACCGGGCCCGAATC | [4] |
| 6 | ICP8 | HSV-1 | AAGCTGGTTGCGTTGGAG | TTTCTGCTGAAGCAGTTCCA | [3] |
| 7 | TK | HSV-1 | ACCCGCTTAACAGCGTCAACA | CCAAAGAGGTGCGGGAGTTT | [3] |
| 9 | VP16 | HSV-1 | TTTGACCCGCGAGATCCTAT | GCTCCGTTGACGAACATGAA | [3] |
| 10 | gC | HSV-1 | GCCCATTTCGTACGACTACA | GGTGCTCTAGAACGGGAATC | [4] |
| 11 | ADAR1p110 | Human | GGCAGCCTCCGGGTG | CTGTCTGTGCTCATAGCCTTGA | [2] |
| 12 | ADAR1p150 | Human | CGGGCAATGCCTCGC | AATGGATGGGTGTAGTATCCGC | [2] |
| 13 | ADAR2 | Human | CGCAGGTTTTAGCTGACGC | GCATCTTTAACATCTGTGCCTGT | [6] |

References

**1.**Cokaric Brdovcak, M., Zubkovic, A., Ferencic, A., Sosa, I., Stemberga, V., Cuculic, D., Rokic, F., Vugrek, O., Hackenberg, M., and Jurak, I. (2018). Herpes simplex virus 1 miRNA sequence variations in latently infected human trigeminal ganglia. Virus Res *256*, 90-95.

2.Cuadrado, E., Booiman, T., van Hamme, J.L., Jansen, M.H., van Dort, K.A., Vanderver, A., Rice, G.I., Crow, Y.J., Kootstra, N.A., and Kuijpers, T.W. (2015). ADAR1 Facilitates HIV-1 Replication in Primary CD4+ T Cells. PLoS One *10*, e0143613.

3.Mattila, R.K., Harila, K., Kangas, S.M., Paavilainen, H., Heape, A.M., Mohr, I.J., and Hukkanen, V. (2015). An investigation of herpes simplex virus type 1 latency in a novel mouse dorsal root ganglion model suggests a role for ICP34.5 in reactivation. J Gen Virol *96*, 2304-2313.

4.Pan, D., Flores, O., Umbach, J.L., Pesola, J.M., Bentley, P., Rosato, P.C., Leib, D.A., Cullen, B.R., and Coen, D.M. (2014). A neuron-specific host microRNA targets herpes simplex virus-1 ICP0 expression and promotes latency. Cell Host Microbe *15*, 446-456.

5.Rutkowski, A.J., Erhard, F., L'Hernault, A., Bonfert, T., Schilhabel, M., Crump, C., Rosenstiel, P., Efstathiou, S., Zimmer, R., Friedel, C.C.*, et al.* (2015). Widespread disruption of host transcription termination in HSV-1 infection. Nat Commun *6*, 7126.

6.Wang, Q., Hui, H., Guo, Z., Zhang, W., Hu, Y., He, T., Tai, Y., Peng, P., and Wang, L. (2013). ADAR1 regulates ARHGAP26 gene expression through RNA editing by disrupting miR-30b-3p and miR-573 binding. RNA *19*, 1525-1536.
